# Supplementary material for: Tuberculosis Treatment Response Monitoring by the Phenotypic Characterization of MTB-Specific CD4+ T-Cells in Relation to HIV Infection Status
Source: Pathogens. 2022 Sep 12;11(9):1034. doi: 10.3390/pathogens11091034 (PMC9506022; doi:10.3390/pathogens11091034)
Supplement: Supplementary file 1 [file pathogens-11-01034-s001.zip › pathogens-1894622-supplementary.pdf]

Supplementary material

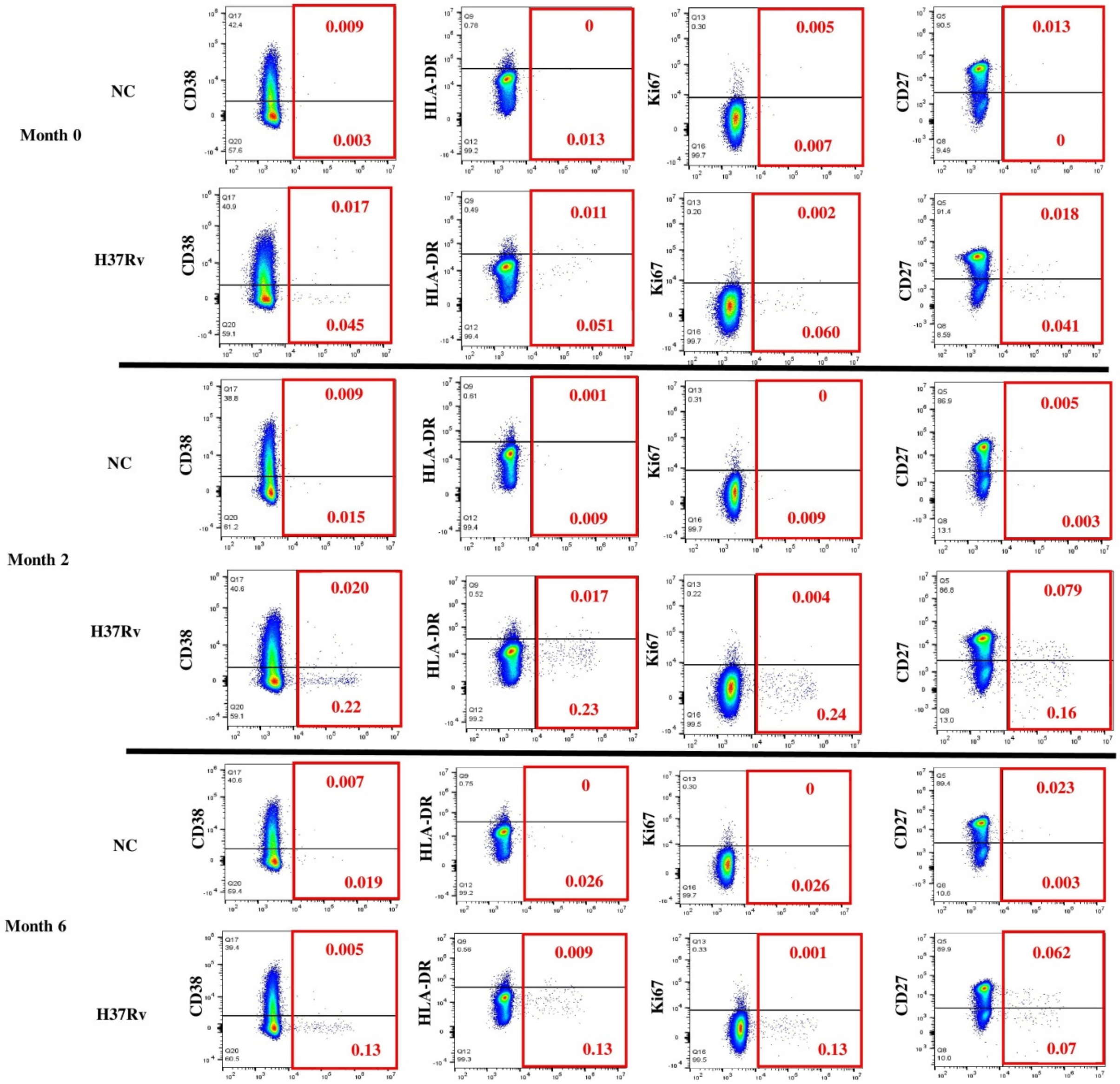

Supplementary Figure S1. Representative dot plots for phenotypic characterization of *MTB*-specific CD4+ T-cells. Shown are dot plots for month 0 or baseline (**upper**), two months into TB treatment (**middle**) and end of TB treatment (**lower**). Dot plots are gated on CD4+ T-cells showing IFNγ (x-axis), activation (CD38, HLA-DR, and Ki67), or maturation (CD27) markers staining (y-axis) without stimulation (NC) and with stimulation (H37Rv). IFNγ+ *MTB*-specific CD4+ T-cells are indicated (red box). The cut-off for the expression of each phenotypic marker is indicated as a black line inside each plot.

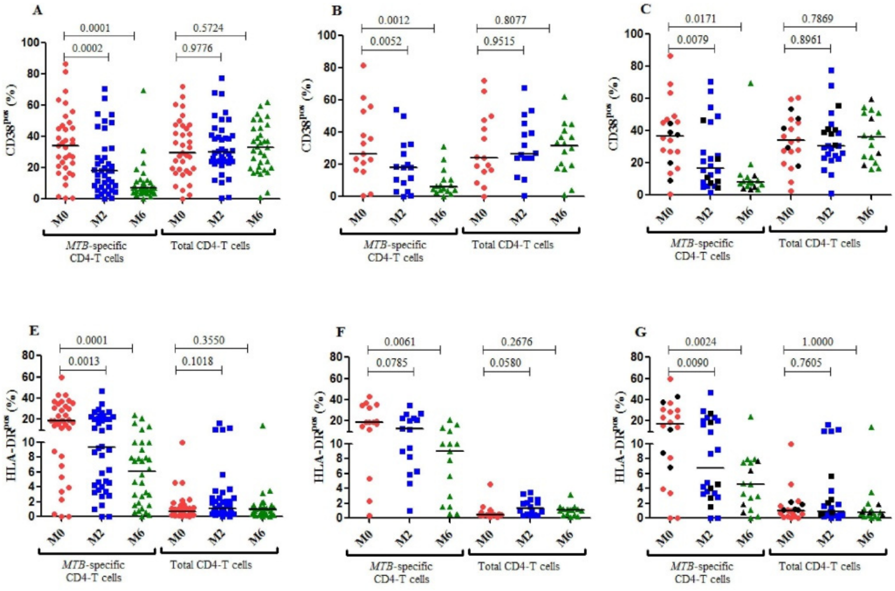

Supplementary Figure S2: Detection of dynamic changes in the expression of the CD38 and HLA-DR markers on *MTB*-specific CD4+ T-cells and Total CD4+T-cells upon TB treatment initiation**.** The frequency of T cells expressing the activation markers CD38 and HLA-DR in all subjects (A, E) and per group: HIV/TB coinfected (C, G) and TB monoinfected (B, F) at BL, M2 and M6 after TB treatment. The red circles, blue squares and green triangles represent baseline, month 2 and 6, respectively. The black symbols in the graphs C and G represent the HIV/TB coinfected subjects that were ART naïve at baseline. *MTB*-specific CD4+ T-cells and total CD4+ T-cells response were characterised after H37Rv stimulation. Bars represent medians. Statistical analyses were performed using Wilcoxon signed rank test for paired samples. P-values are indicated.

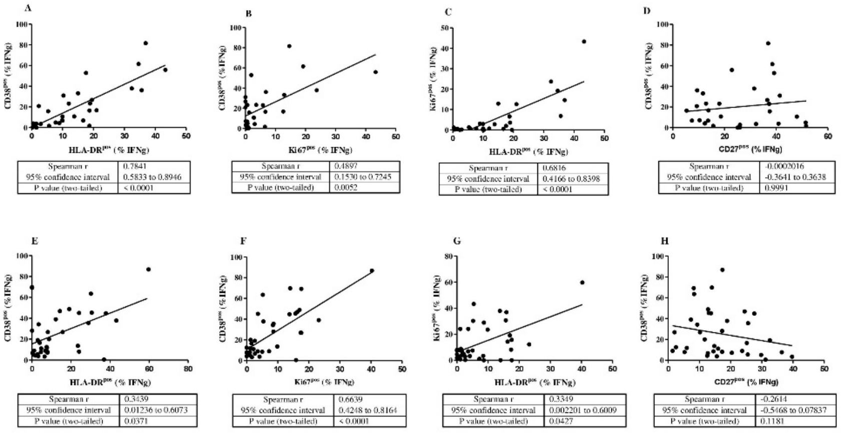

Supplementary Figure S3: Correlation analysis of activation and maturation markers expression on *MTB*-specific CD4+ T-cells before and after TB treatment (n=70). The frequencies of *MTB*-specific CD4+ T-cells expressing activation or maturation markers after stimulation were plotted for CD38 and HLA-DR (**A, E**),

CD38 and Ki67 (**B, F**), Ki67 and HLA-DR (**C, G**), CD38 and CD27 (**D, H**) in TB monoinfected subjects (n=15) and HIV/TB coinfected (n=20) on the y- and x-axis. Graphs A-D and E-F represent subjects TB monoinfected and HIV/TB coinfected, respectively. Spearman's rank test was used for statistical analysis. P value is indicated.

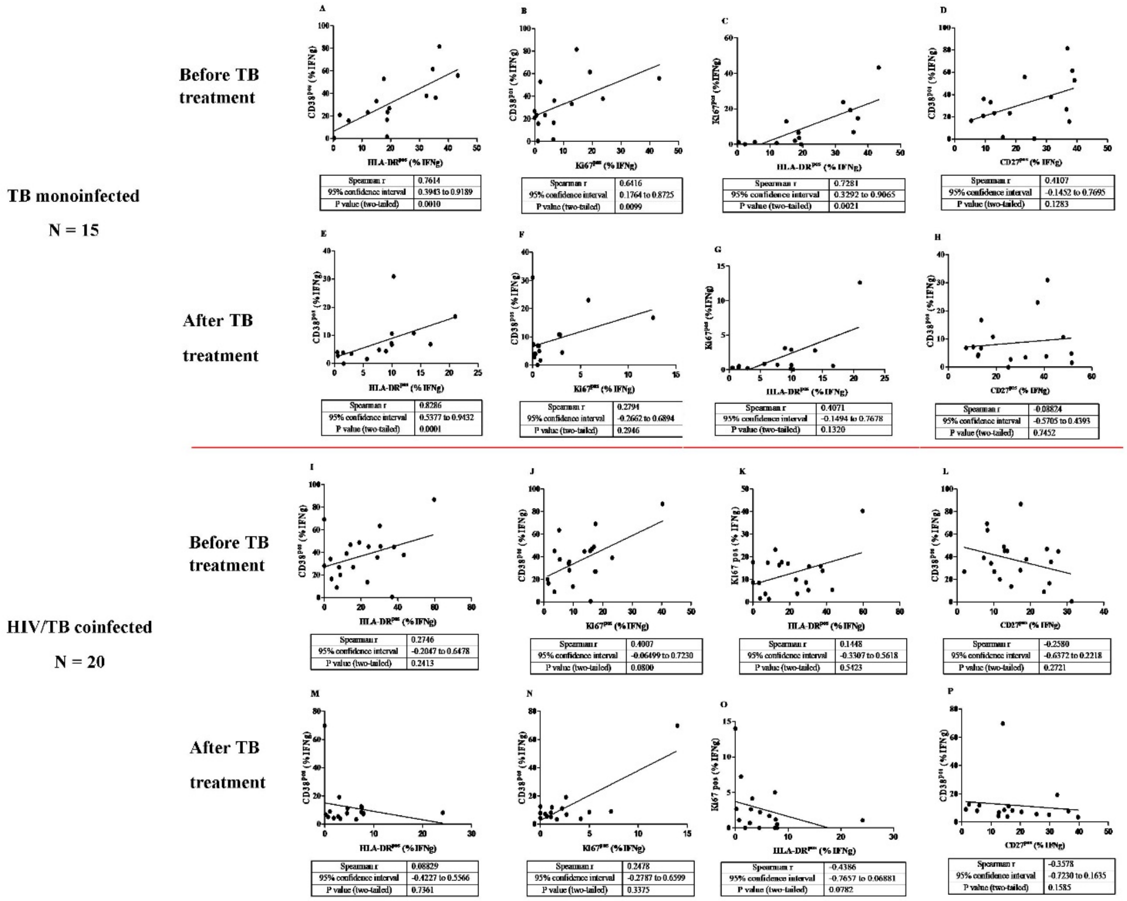

Supplementary Figure S4: Correlation analysis of activation and maturation markers expression on *MTB*-specific CD4+ T-cells before and after TB treatment, separately. The frequencies of *MTB*-specific CD4+ T-cells expressing activation or maturation markers after stimulation were plotted for CD38 and HLA-DR (**A, E, I, M**), CD38 and Ki67 (**B, F, J, N**), Ki67 and HLA-DR (**C, G, K, O**), CD38 and CD27 (**D, H, L, P**) in subjects TB monoinfected (n=15) and HIV/TB coinfected (n=20) on the y- and x-axis. Graphs A-H and I-P represent subjects TB monoinfected and HIV/TB coinfected, respectively. Spearman's rank test was used for statistical analysis. P value is indicated.

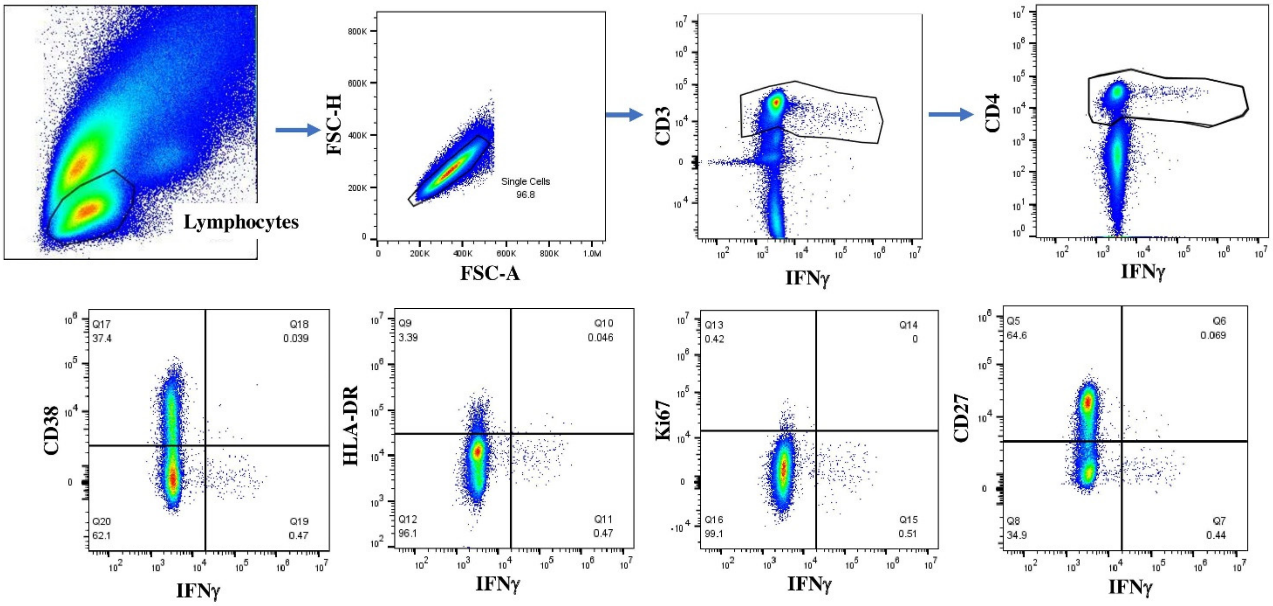

Supplementary Figure S5. Gating strategy for *MTB*-specific CD4+ T-cells producing IFNγ◎T-cells producing IFNan’s rank test was used for

Supplementary Table S1. Percentage of responders per antigen in each time point, in HIV/TB coinfected and TB monoinfected study groups. The samples were stimulated with H37Rv whole cell lysate (H37Rv), Purified Protein Derivative (PPD) and ESAT6/ CFP10 (E6C10) antigens. The percentage of responders was determined per time point and an average calculated per antigen based on total number of subjects tested in each study group.

	TB^+					HIV^+/TB^+				
	Time point					Time point				
Antigen	0	2	6	Average of responders (%)	Total of tested (n)	0	2	6	Average of responders (%)	Total of tested (n)
H37Rv (%)	15 (88.2)	16 (94.1)	16 (94.1)	92.2	17	21 (77.8)	24 (88.9)	18 (66.7)	78.8	27
PPD (%)	11 (64.7)	15 (88.2)	16 (94.1)	82.4	17	13 (48.2)	22 (81.5)	16 (59.3)	62.9	27
E6C10 (%)	5 (29.4)	10 (58.8)	10 (58.8)	49.0	17	14 (51.9)	20 (74.0)	11 (40.7)	55.6	27
